# A self-assembled nanoparticle vaccine displaying chimeric and trimeric RBD-HRC elicits broad-spectrum neutralizing antibodies against multiple coronaviruses

**DOI:** 10.1128/spectrum.03797-25

**Published:** 2026-03-30

**Authors:** Didi Wan, Lili Li, He Feng, Mengyu Wang, Shuo Jia, Pengyu Zhang, Jiaxu Wang, Junxia Yang, Yijie Zhang, Boya Ji, Yushun Wan, Zhengkun Xie, Jian Shang

**Affiliations:** 1 BGI College & Henan Institute of Medical and Pharmaceutical Sciences, Zhengzhou University 668040, Zhengzhou, Henan, China; 2 College of Life Sciences, Henan Normal University 66519, Xinxiang, Henan, China; 3 College of Basic Medicine, Chongqing Medical University 12550, Chongqing, Chongqing, China; 4 College of Chemistry, Zhengzhou University 648401, Zhengzhou, Henan, China; 5 State Key Laboratory of Metabolic Dysregulation & Prevention and Treatment of Esophageal Cancer, School of Life Sciences, Zhengzhou University 12636, Zhengzhou, Henan, China; The University of Texas Medical Branch at Galveston, Galveston, Texas, USA

**Keywords:** nanoparticle, broad-spectrum, vaccine design, coronaviruses

## Abstract

Coronaviruses, known for their high mutation rates and character of cross-species transmission, continue to pose significant threats to global health, as demonstrated by outbreaks of SARS, MERS, and SARS-CoV-2. Vaccines remain the most effective means of preventing viral infection, transmission, and epidemic spread. However, traditional vaccine development faces significant challenges due to the rapid evolution of viruses and their ability to evade the immune system. In response, we present a novel vaccine design based on self-assembling nanoparticles that display multiple glycosylation-modified receptor-binding domain-heptad repeat C-domain (RBD-HRC) trimers, forming a multimerized, polyvalent chimeric RBD-HRC vaccine. This approach enhances immunogenicity by closely mimicking the native structure of the spike protein, thereby eliciting broad-spectrum neutralizing antibodies that can target coronavirus-derived pseudoviruses from different genera. Notably, it even demonstrates neutralizing activity against coronaviruses not included in the vaccine design. Additionally, this vaccine formulation induces a robust cellular immune response. The multivalent nature of the nanoparticle vaccine promotes stronger T-cell activation and exhibits enhanced broad-spectrum activity. Our strategy offers a scalable and versatile platform for the development of vaccines against a wide range of viral pathogens.

**IMPORTANCE:**

Broad-spectrum vaccines are urgently needed to control the rapid evolution of viruses and their cross-species transmission. The effective presentation of epitopes, especially conserved ones, plays a critical role in vaccine design. Moreover, the polymerization of epitopes has been shown to significantly enhance immunogenicity. In this study, we present a scalable platform for developing broad-spectrum vaccines against rapidly evolving pathogens. This vaccine platform, based on self-assembling nanoparticles, displays glycosylation-modified coronavirus spike receptor-binding domain-heptad repeat C-domain (RBD-HRC) trimers, enabling the formation of multimerized and polyvalent chimeric antigens. We found that the trivalent RBD-HRC nanoparticle vaccine elicits the broadest neutralizing antibody response, strongest T-cell activation, and highest neutralization potency compared to other formulations, offering valuable insights for future vaccine development.

## INTRODUCTION

Emerging and re-emerging infectious viral diseases continue to pose a major threat to global public health (1). Vaccines remain the primary and most effective strategy for preventing viral infection and transmission (2–4). Since the outbreak of the COVID-19 pandemic, vaccine development, particularly mRNA vaccines, has achieved unprecedented breakthroughs, largely driven by advances in mRNA modification technologies (5, 6). However, the ongoing global pandemic and continuous evolution of SARS-CoV-2 have reduced the effectiveness of existing vaccines, leading to breakthrough infections and sustained transmission (7, 8). Meanwhile, numerous sarbecoviruses and merbecoviruses have been identified in wildlife, raising concerns about their potential to spill over into human populations and threaten public health (9–14). In this context, the development of broad-spectrum antiviral strategies has become critically important, with broad-spectrum vaccines playing a particularly vital role.

The spike protein plays a key role in mediating coronavirus entry into host cells (15). It serves as the primary antigen that stimulates the production of neutralizing antibodies, with approximately 90% of these antibodies targeting the receptor-binding domain (RBD) (16–18). Therefore, the RBD is considered an ideal target for vaccine development and has played a crucial role in the prevention and control of the COVID-19 pandemic (19). However, as the virus continues to mutate, the effectiveness of the original vaccines has declined (20, 21). Designing and developing new broad-spectrum vaccines is therefore essential, not only to address currently circulating variants but also to prevent the cross-species transmission of potentially dangerous zoonotic coronaviruses.

Compared to the full spike protein, the standalone RBD exposes some non-neutralizing epitopes (22, 23). Introducing glycosylation sites to mask these non-neutralizing regions has been shown to enhance the efficacy of RBD-based vaccines (24). Dimerization of the RBD can increase neutralizing antibody titers by 10- to 100-fold (25), and trimerization using the conserved heptad repeat C-domain (HRC) can induce high-titer, broad-spectrum neutralizing antibodies as well as robust T-cell responses (26). These findings suggest that multimerization of the RBD can elicit stronger immune responses, opening the possibility of developing polyvalent RBD vaccines derived from different coronaviruses to provide broader protective immunity.

In this study, we propose an innovative strategy: displaying multiple glycosylation-modified RBD-HRC trimers on an I53-DN5A/B nanoparticle, which has previously been shown to be effective for presenting protein trimers, to create a multimerized, polyvalent chimeric RBD-HRC vaccine capable of inducing stronger and broader neutralizing antibodies. Our research presents a novel approach for broad-spectrum vaccine design and lays a critical technical foundation for preventing the emergence of future coronavirus variants.

## RESULTS

### Design and self-assembly of nanoparticle-conjugated antigens

**Fig 1:**
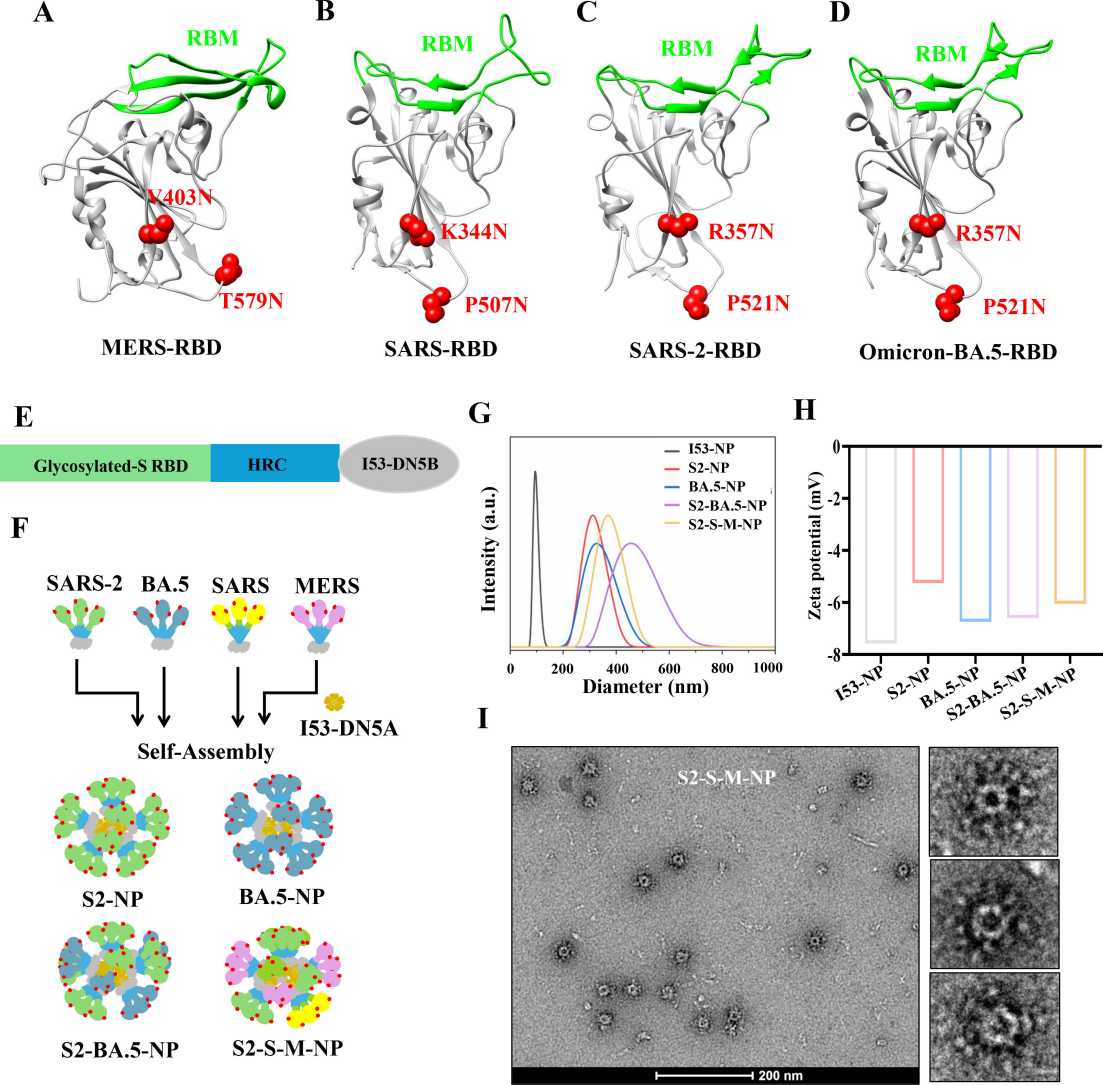
Construction and characterization of RBD-HRC nanoparticle vaccines. (**A**) The structure highlights the glycosylation sites (V403N and T579N, shown in red) that were previously introduced to mask non-neutralizing epitopes on the MERS RBD. RBD: receptor-binding domain; RBM: receptor-binding motif. (**B–D**) The corresponding glycosylation sites are introduced into the RBDs of SARS (K344N and P507N), SARS-CoV-2 (R357N and P521N), and Omicron (R357N and P521N) to mask non-neutralizing antigens. (**E**) Schematic drawing of the glycan-modified RBD-HRC-I53-DN5B vaccine design. HRC: the C-terminal heptad repeat domain; I53-DN5B: the one part of the I53 nanoparticle. (**F**) The self-assembly of RBD-HRC nanoparticles. (**G**) Dynamic light scattering (DLS) was used to analyze the size distribution of I53 and the RBD-HRC nanoparticles. (**H**) The zeta potential distribution of I53 and the RBD-HRC nanoparticles was evaluated. (**I**) Negative-stain electron microscopy was employed to visualize the purified trivalent nanoparticle vaccine. S2-NP: SARS-CoV-2 RBD-HRC nanoparticles; BA.5-NP: Omicron BA.5 RBD-HRC nanoparticles; S2-BA.5-NP: a divalent nanoparticle vaccine composed of SARS-CoV-2 and BA.5 RBD-HRCs; S2-S-M-NP: a trivalent nanoparticle vaccine composed of SARS-CoV-2, SARS, and MERS RBD-HRCs.

To enhance broad-spectrum efficacy, we selected the receptor-binding domains (RBDs) from the prototypical SARS-CoV-2 strain and an Omicron variant, BA.5, as well as from two other highly pathogenic β-coronaviruses, SARS-CoV and MERS-CoV. To improve immunogenicity, all RBDs were modified by introducing two N-glycosylation sites to mask non-neutralizing epitopes. The introduced mutation sites, K344N and P507N for SARS-CoV RBD, R357N and P521N for SARS-CoV-2 prototype and BA.5 RBD, were selected structurally corresponding to MERS-CoV RBD (Fig. 1A through D), which has been previously reported (27). These modifications follow the canonical N-glycosylation motif “N-X-T/S.”

Furthermore, to enhance the broad-spectrum and immunogenicity, we fused a relatively conserved HRC domain to the C-terminus of RBD, which not only facilitates trimer formation but also acts as an antigen to induce broadly neutralizing antibodies. The RBD-HRC construct mimics a small trimeric S protein, effectively clustering the major antigenic sites. To further enhance immunogenicity and facilitate the development of multivalent vaccines, we appended the I53-DN5B component to the C-terminus of HRC (Fig. 1E). This modification enables the protein to self-assemble with I53-DN5A, forming nanoparticles that display the trimeric antigen. By varying the source of RBD during nanoparticle assembly, different multivalent vaccines can be generated. In this study, we produced and purified a total of four nanoparticle vaccine formulations, displaying: (i) glycosylated trimeric prototype SARS-CoV-2 RBD, (ii) Omicron BA.5 RBD, (iii) a bivalent vaccine containing both SARS-CoV-2 prototype and BA.5 RBDs, and (iv) a trivalent nanoparticle vaccine incorporating RBDs from SARS-CoV-2, SARS-CoV, and MERS-CoV (Fig. 1F; Fig. S1D), representing different antigenic combinations.

To evaluate the self-assembly effectiveness of the nanoparticle vaccine, we first employed a Nanoparticle Size and Zeta Potential Analyzer to measure the particle size and zeta potential of the purified vaccine. The results revealed that, compared to the individual I53 nanoparticles, the nanoparticle vaccine exhibited a significant increase in particle size (*P* < 0.001) (Fig. 1G), while the zeta potential remained largely unchanged (*P* > 0.05) (Fig. 1H). This suggests that the glycan-modified RBD-HRC was successfully displayed on the surface of the nanoparticles and that the structural integrity of the nanoparticle vaccine was maintained. Additionally, we characterized the nanoparticle vaccine using negative-stain transmission electron microscopy. The images showed that the nanoparticle vaccine was fully assembled, with the trimeric RBD-HRC antigen uniformly displayed on its surface (Fig. 1I; Fig. S1H). This further confirms that our nanoparticle vaccine was both structurally and assembly-wise successful.

### Nanoparticle vaccines elicit potent antigen-specific antibody responses in mice

**Fig 2:**
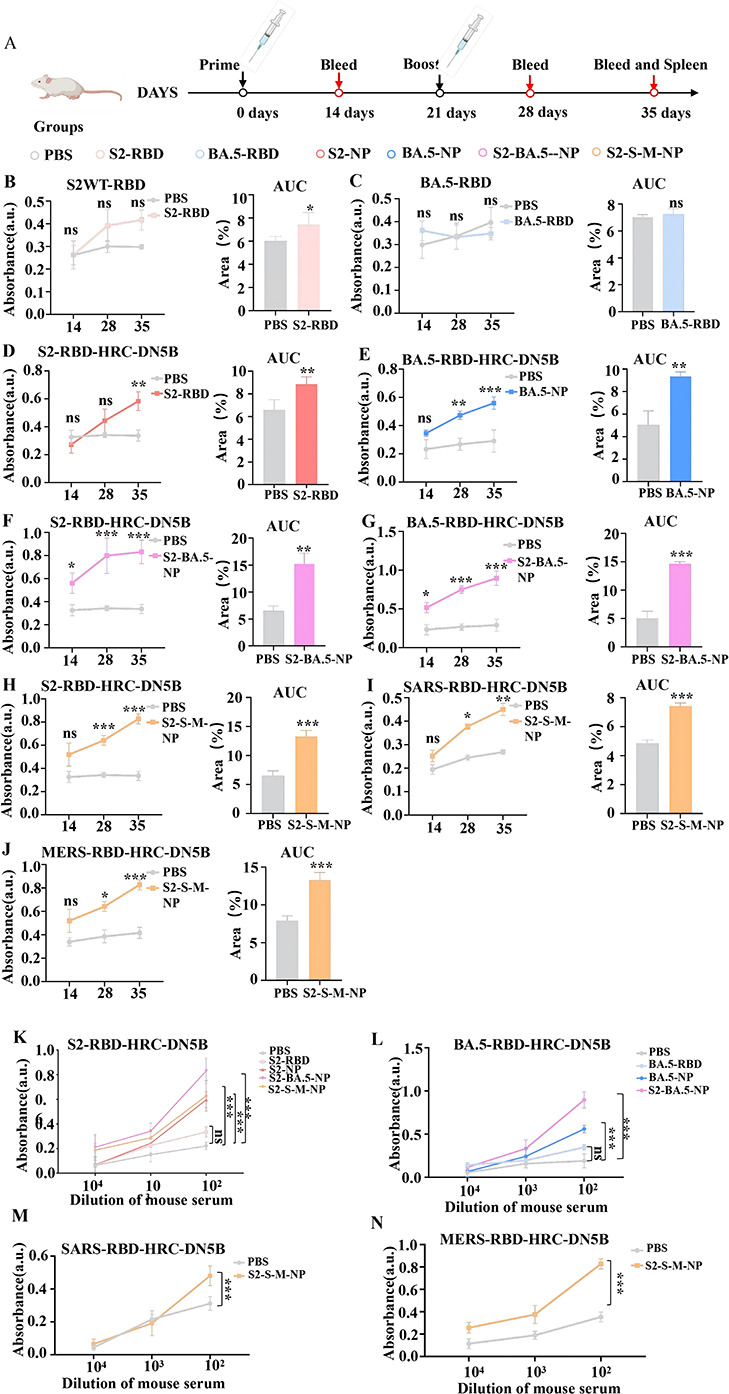
Immunization with RDB-HRC nanoparticles elicits a robust humoral response in mice. (**A**) The immunization protocol in mice is outlined. Six-week-old female BALB/c mice were immunized with 10 μg of mosaic nanoparticles, while the control group received PBS. Mice received a booster dose 3 weeks after the initial vaccination. (**B–J**) Total IgG levels in the sera of vaccinated mice were measured 14, 28, and 35 days after the primary vaccination using ELISA to assess binding to SARS-CoV-2-WT-RBD, SARS-CoV-2-BA.5-RBD, SARS-CoV-2-WT-RBD-HRC, SARS-CoV-2-BA.5-RBD-HRC, MERS-CoV-RBD-HRC, and SARS-CoV-RBD-HRC, respectively. Each group included *n* = 5 mice. Each panel contains two subplots: the left subplot shows AU obtained at a serum dilution of 1:100 for each time point, and the right subplot presents the AUC values integrated from the AU data of the three time points (14, 28, and 35 days). (**K–N**) The IgG dilution ratio in sera from vaccinated mice was evaluated using ELISA on day 35 post-primary vaccination. BA.5-NP: Omicron BA.5 RBD-HRC nanoparticles; S2-BA.5-NP: a divalent nanoparticle vaccine composed of SARS-CoV-2 and BA.5 RBD-HRCs; S2-NP: SARS-CoV-2 RBD-HRC nanoparticles; S2-S-M-NP: a trivalent nanoparticle vaccine composed of SARS-CoV-2, SARS, and MERS RBD-HRCs. All data are shown as mean ± SEM (*n* = 5). Statistical significance was determined using a *t*-test, with significance levels indicated as follows: *P < 0.05, **P < 0.01, and ***P < 0.001.

To assess the immunogenicity of all self-assembled nanoparticle vaccines, we first evaluated the antibody responses elicited by these vaccines. Female BALB/c mice were immunized via subcutaneous injection using a prime-boost regimen (days 0 and 21) with either 10 µg of monomeric RBD, used as a monomeric antigen control, the self-assembled RBD-HRC trimeric nanoparticles, or phosphate-buffered saline (PBS) as a negative control, in the absence of any adjuvant. Serum samples were collected on days 14, 28, and 35 following the initial immunization (Fig. 2A), and antigen-specific IgG levels in the sera were detected by indirect enzyme-linked immunosorbent assay (ELISA). To enhance the robustness of the quantitative analysis of antibody responses, we analyzed not only the absorbance values (AU) at each individual time point but also calculated the area under the curve (AUC) for each sample by integrating the AU data across the three measured time points (14, 28, and 35 days). The AUC provides a composite measure of the antigen-specific IgG response dynamics over the entire immunization period, offering a more robust basis for inter-group comparisons.

The kinetics of antigen-specific IgG antibody titers and subclass profiles in the sera of immunized mice were then assessed by indirect ELISA. Monomeric SARS-CoV-2 and BA.5 RBDs failed to elicit detectable antigen-specific IgG responses (*P* > 0.05 compared to PBS), highlighting their dependence on adjuvants. In contrast, all nanoparticle vaccines induced progressively increasing antibody levels after the booster dose, with no plateau reached by the end of the study (day 35) (Fig. 2B through 2J). Notably, the bivalent (S2-BA.5-NP) and trivalent (S2-S-M-NP) nanoparticle vaccines elicited significantly higher antibody titers than the mono-antigen nanoparticle vaccines (*P* < 0.01) (Fig. 2K through 2N), suggesting that antigenic synergy enhances immunogenicity.

Additionally, the antibody subclasses induced by all vaccines were analyzed separately. Except for serum induced by the monomeric RBDs, all sera induced by nanoparticle vaccines showed a sustained increase in IgG1 titers after the booster, with endpoint titers more than 100-fold higher than those in the PBS group (*P* < 0.001) (Fig. S2I). IgG2a titers exhibited a similar kinetic trend, with their levels being markedly lower than those of IgG1 (Fig. S2R). The ratio of IgG1/IgG2a exceeded 1 across all nanoparticle vaccines, with this tendency becoming more pronounced as vaccine valency increased (Fig. S2S), indicating a strong Th2-skewed immune response. This Th2-biased profile suggests that vaccine-induced protection may rely on antibody-mediated mechanisms, such as antibody-dependent cellular phagocytosis (ADCP), which differs from the predominantly cell-mediated immunity typically induced by viral vector vaccines.

### Nanoparticle vaccines induce cellular immune responses

Cellular immunity, mediated by both CD4^+^ and CD8^+^ T cells, plays a critical role in defending against pathogen infections (19). Robust CD4^+^ T helper cell responses are crucial for generating high-titer antibody responses. In patients with COVID-19, SARS-CoV-2-specific CD4^+^ and CD8^+^ T-cell responses have been shown to correlate strongly with the production of neutralizing antibodies (28, 29). Therefore, we investigated the capacity of nanoparticle vaccines to activate T-cell responses. For this purpose, spleens were isolated from immunized mice 35 days after primary immunization to prepare single-cell suspensions.

**Fig 3:**
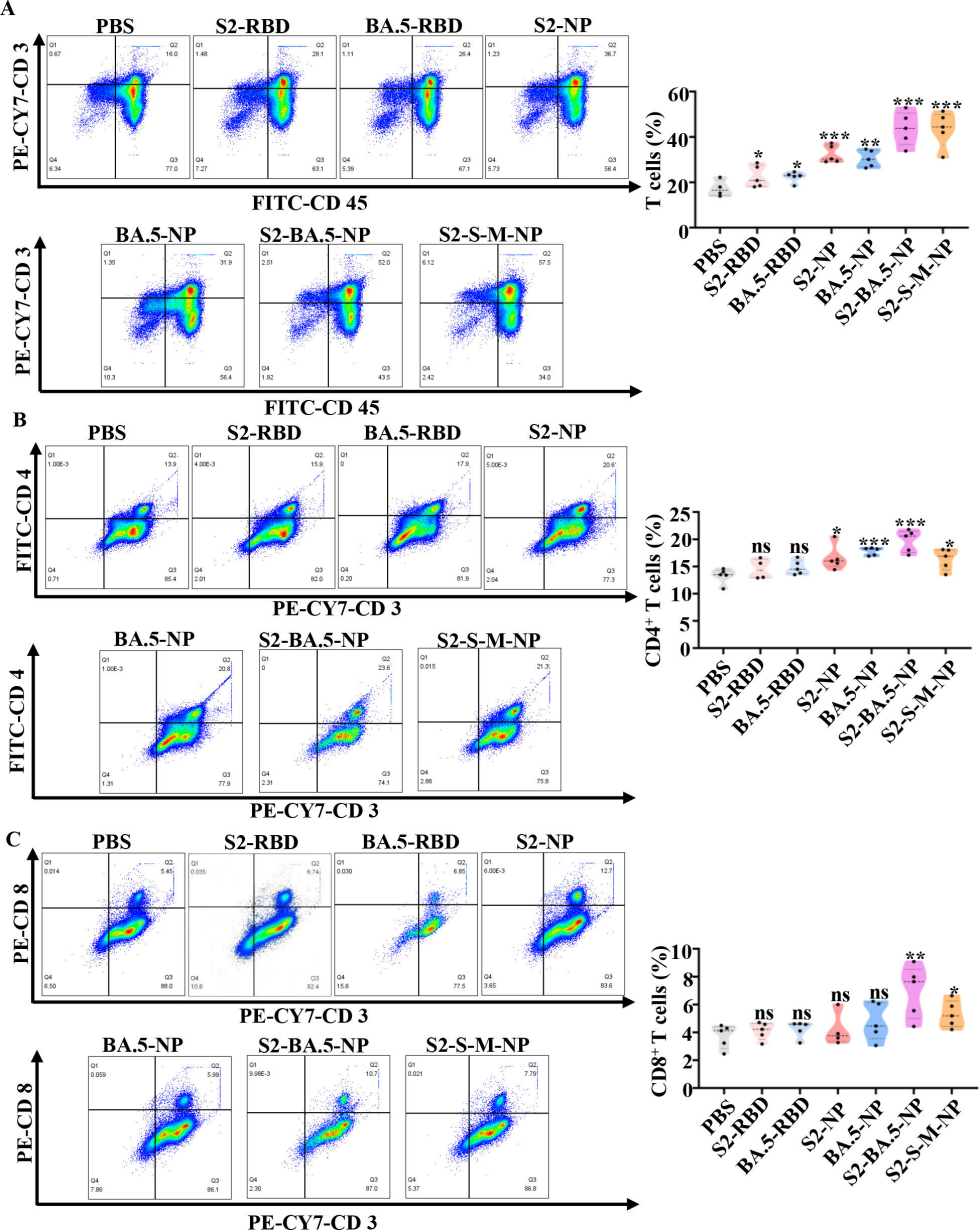
RBD-HRC nanoparticles elicit a strong cellular immune response in mice. (**A–C**) Six-week-old female BALB/c mice were immunized with 10 μg of the respective nanoparticle vaccines, while the control group received PBS. Spleens were collected 14 days after the booster immunization. The frequencies of T cells (**A**), CD4^+^ T cells (**B**), and CD8^+^ T cells (**C**) were analyzed by flow cytometry. S2-NP: SARS-CoV-2 RBD-HRC nanoparticles; BA.5-NP: Omicron BA.5 RBD-HRC nanoparticles; S2-BA.5-NP: a divalent nanoparticle vaccine composed of SARS-CoV-2 and BA.5 RBD-HRCs; S2-S-M-NP: a trivalent nanoparticle vaccine composed of SARS-CoV-2, SARS, and MERS RBD-HRCs. Data are shown as mean ± SD. Statistical significance is indicated as follows: ns, not significant; *P < 0.05, **P < 0.01, ***P < 0.001, and ****P < 0.0001.

Splenocytes were first stained with FITC-CD45 and PE-Cy7-CD3 antibodies and analyzed by flow cytometry to determine the proportion of mature T lymphocytes (CD3^+^). Nanoparticle vaccine-immunized groups exhibited an increase in splenic mature CD3^+^ T-cell proportions compared to monomeric RBD and PBS control groups (*P* < 0.01). Notably, multi-antigen nanoparticle vaccines induced significantly stronger mature T-cell activation than mono-antigen groups, indicating antigen diversity enhances T-cell repertoire recruitment efficiency (Fig. 3A).

Splenocytes were then stained with PE-Cy7-conjugated CD3 and PE-conjugated CD4 antibodies to quantify CD4^+^ T lymphocyte subsets. Consistent with the overall trend observed in mature T cells, the nanoparticle vaccines induced significantly higher percentages of splenic CD4^+^ T cells compared to the monomeric RBD group. Notably, the bivalent nanoparticle vaccine induced a greater proportion of CD4^+^ T cells than any of the mono-antigen groups and the trivalent nanoparticle vaccine (Fig. 3B).

To assess cytotoxic T-cell responses, splenocytes were stained with PE-Cy7-conjugated CD3 and PE-conjugated CD8 antibodies. The results indicated that neither the monomeric RBD nor the mono-antigen nanoparticle vaccines induced a significant increase in CD8^+^ T-cell proportions (*P* > 0.05), while the multi-antigen nanoparticle vaccines nearly doubled the proportion of CD8^+^ T cells (Fig. 3C). This suggests that the multivalent form of the vaccine is more conducive to the activation of CD8^+^ T cells.

### Nanoparticle vaccines elicit broad-spectrum neutralizing antibodies

**Fig 4:**
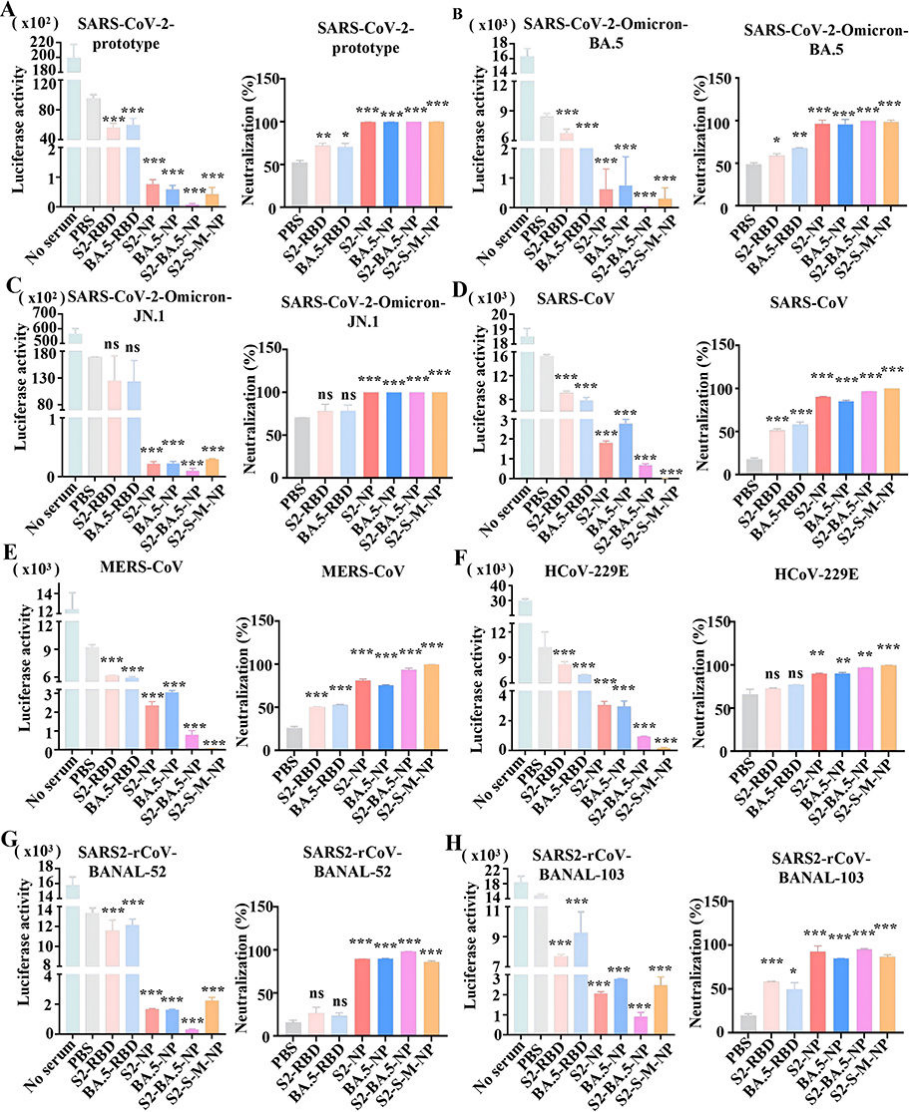
RBD-HRC nanoparticles induce broad-spectrum neutralizing activity. For each pseudovirus, neutralization data are presented in two complementary formats within the same panel. The left graph displays raw infectivity as RLU. The right graph displays the corresponding percent neutralization, calculated from the RLU data as described in the Materials and Methods. (**A–C**) Neutralization titers of immunized serum against the SARS-CoV-2 prototype and its variants Omicron BA.5 and JN.1 were determined using S-pseudotyped viruses. (**D–F**) Neutralization titers against SARS-CoV-1, MERS-CoV, and HCoV-229E were evaluated by pseudovirus assay. (**G–H**) Neutralization titers against SARS-CoV-2-related bat coronaviruses BANAL-52 and BANAL-103 were measured using a pseudovirus assay. S2-NP: SARS-CoV-2 RBD-HRC nanoparticles; BA.5-NP: Omicron BA.5 RBD-HRC nanoparticles; S2-BA.5-NP: a divalent nanoparticle vaccine composed of SARS-CoV-2 and BA.5 RBD-HRCs; S2-S-M-NP: a trivalent nanoparticle vaccine composed of SARS-CoV-2, SARS, and MERS RBD-HRCs. All data are presented as mean ± SD, with statistical significance denoted as follows: ns, not significant; *P < 0.05, **P < 0.01, and ***P < 0.001.

To further evaluate the spectrum of neutralizing antibodies elicited by the nanoparticle vaccines, a pseudovirus-based neutralization assay was employed. The neutralization data for each pseudovirus in Fig. 4 are presented in a dual-panel format: the left graph of each subfigure displays the raw infectivity readout as relative luminescence units (RLU), while the right graph displays the corresponding percent neutralization calculated from the same RLU data set. This presentation facilitates the comparison of neutralization breadth and potency elicited by the different nanoparticle vaccine designs against each coronavirus. Immunized sera were collected from all experimental groups via retro-orbital plexus bleeding 35 days after the initial immunization. The respective coronavirus spike-packaged pseudoviruses were first incubated with the sera for 1 h at 37°C, and the mixtures were then used to infect 293T cells stably expressing human ACE2 (293T-hACE2), human DPP4 (293T-hDPP4), or human APN (293T-hAPN). The efficiency of pseudovirus entry was determined by measuring luciferase activity in lysates from the infected cells, which reflected the neutralizing capacity of the sera.

First, we evaluated the neutralizing activity of the sera against pseudoviruses of the SARS-CoV-2 prototype and its variants, including Omicron BA.5 and JN.1. The sera from mice immunized with monomeric RBD showed moderate neutralizing activity against the pseudoviruses of the SARS-CoV-2 prototype (Fig. 4A) and BA.5 (Fig. 4B), but no neutralization against JN.1 pseudoviruses (Fig. 4C), indicating that monomeric RBD alone, without adjuvant, is insufficient to elicit strong neutralizing antibodies or broad protective immunity. In contrast, sera from mice immunized with nanoparticle vaccines displayed clear neutralizing activity against pseudoviruses derived from both the SARS-CoV-2 prototype and Omicron variants, with significantly higher potency compared to monomeric RBD. Notably, the strongest neutralizing activity was observed in sera from mice immunized with nanoparticles displaying trimeric RBD-HRC derived from both the SARS-CoV-2 prototype and BA.5 (S2-BA.5-NP) (Fig. 4A through C).

Given that the vaccine design incorporates a conserved HRC domain, it is expected to induce broad-spectrum antibodies. Additionally, antigen multimerization has been reported to enhance the induction of such antibodies (30, 31). To test this, we conducted neutralization assays using pseudoviruses of SARS-CoV and MERS-CoV—two other highly pathogenic β-coronaviruses. The results showed that sera from mice immunized with monomeric RBD exhibited some neutralizing activity against both SARS-CoV and MERS-CoV pseudoviruses (Fig. 4D and E), suggesting potential cross-protective effects among coronaviruses. Notably, sera from mice immunized with nanoparticle-based vaccines showed significantly higher neutralization titers against SARS-CoV and MERS-CoV pseudoviruses compared to those immunized with monomeric RBD. Furthermore, sera from mice receiving bivalent and trivalent nanoparticle vaccines demonstrated enhanced neutralization against both viruses compared to those receiving mono-antigen nanoparticle vaccines, with the trivalent nanoparticle vaccine eliciting the strongest neutralizing response against SARS-CoV and MERS-CoV pseudoviruses (Fig. 4D and E).

We next performed a neutralization assay using pseudoviruses of human coronavirus 229E from the α-genus, which was not included in the vaccine design of this study, to assess the potential breadth of the antibody response induced by the vaccines. While hCoV-229E utilizes APN as its receptor and differs substantially in sequence and structure from the targeted betacoronaviruses, we observed notable neutralization activity. The neutralization results were highly similar to those observed for SARS-CoV and MERS-CoV pseudoviruses. The trivalent nanoparticle vaccine induced the strongest neutralizing activity, while the bivalent nanoparticle vaccine showed a more robust neutralizing response against hCoV-229E pseudoviruses compared to the mono-antigen nanoparticle vaccines (Fig. 4F). This observed activity, although its precise mechanism requires further investigation, suggests that the multimerized nanoparticle formulation containing conserved HRC domains may elicit antibodies capable of targeting shared or structurally convergent epitopes involved in viral entry, even across coronavirus genera.

Coronaviruses circulating in nature pose a significant threat to human health. To further evaluate the neutralizing effect of sera from our nanoparticle vaccine against bat coronaviruses, we used pseudoviruses of two SARS-CoV-2-related bat coronaviruses, BANAL-52 and BANAL-103, as representatives. The monomeric RBD groups showed minimal neutralizing activity against both pseudoviruses. However, sera from all nanoparticle vaccine-immunized groups effectively neutralized both bat-derived pseudoviruses. Notably, the bivalent nanoparticle vaccine containing SARS-CoV-2 prototypic RBD and BA.5 RBD exhibited significantly higher neutralizing titers against BANAL-52 and BANAL-103 compared to other nanoparticle vaccines (Fig. 4G and H). This difference may be attributed to the high sequence homology between BANAL-52/-103 and the SARS-CoV-2 spike protein.

## DISCUSSION

Coronaviruses continue to present significant health risks to humans through cross-species transmission and mutation-driven immune evasion (32–34). Vaccination remains the primary and most effective strategy for preventing and controlling viral outbreaks, although effectiveness varies considerably across different viruses (1, 35). Numerous vaccines targeting the original SARS-CoV-2 strain have received emergency use authorization globally (36–38). Phase III clinical trials have reported protective efficacies ranging from 66.1% to 95% after administration of the second dose (39, 40). However, the emergence of antigenically distinct variants with reduced susceptibility to existing vaccines has become a major obstacle to global pandemic control (41–46). Studies show a decline in the neutralizing activity of both vaccine-induced and convalescent antibodies against SARS-CoV-2 variants, particularly the Beta (B.1.351) (40–42) and Omicron (B.1.1.529) (47–49) lineages. These findings highlight the limitations of current monovalent, strain-specific vaccine strategies in keeping pace with coronavirus evolution, underscoring an urgent need to transition toward the development of broad-spectrum vaccines.

Compared to the full-length spike protein, the RBD contains the majority (>90%) of neutralizing antibody epitopes (17, 18). However, it struggles to independently elicit a robust immune response and may expose certain non-neutralizing epitopes (50, 51). Previous studies have demonstrated that enhancing RBD immunogenicity can be achieved by introducing glycosylation sites to mask non-neutralizing epitopes and through multimerization (25, 52–54). Leveraging bioinformatics and glycoengineering approaches, we optimized the RBDs of SARS-CoV-2 prototype, Omicron BA.5, SARS-CoV, and MERS-CoV based on structural insights and fused them with a conserved HRC domain to generate stable trimeric RBD-HRC complexes that closely resemble the native viral spike protein. This engineered design reduces the exposure of non-neutralizing epitopes compared to full-length spike proteins, thereby substantially improving immunogenic precision.

Furthermore, an I53 self-assembling nanoparticle was employed to present the trimeric RBD-HRC, thereby promoting the development of multivalent nanoparticle vaccines. *In vivo* immunogenicity evaluations indicated that these multivalent nanoparticle vaccines generated significantly stronger and more sustained antigen-specific IgG responses compared to monomeric RBD or single-antigen nanoparticle formulations. Flow cytometric analysis further demonstrated enhanced activation of both CD4^+^ and CD8^+^ T cells, with a Th2-skewed IgG1/IgG2a ratio exceeding 1.5, aligning with established vaccine profiles (55). Importantly, the multi-antigen formulation resulted in a marked increase in CD8^+^ T-cell proportions, suggesting that this platform is particularly effective in stimulating cytotoxic T lymphocyte responses. This coordinated induction of both potent humoral and robust cellular immunity constitutes a key immunogenic characteristic of our vaccine design. We hypothesize that the dense and repetitive antigen array on the nanoparticle surface not only augments B cell receptor engagement, leading to superior antibody responses (56, 57), but also facilitates more efficient antigen cross-presentation via the MHC-I pathway, thereby activating CD8^+^ T cells (58). This synergy—where robust CD4^+^ T-cell assistance supports the development of high-affinity antibodies and memory B cells (59), while activated CD8^+^ T cells potentially aid in viral clearance (60, 61)—indicates that our vaccine may elicit a balanced and comprehensive protective response. Consequently, the synergistic antigen arrangement on the nanoparticle scaffold not only enhances immunogenicity but also provides a structural basis for inducing both cross-reactive antibody responses and a favorable cell-mediated immunity profile, a combination that may be critical for achieving durable and broad protection. Future challenge studies will be crucial to functionally validate the protective contribution of each immune component.

Importantly, the multivalent nanoparticle vaccines exhibited higher neutralizing titers against pseudoviruses of both the SARS-CoV-2 prototype and Omicron BA.5 variant compared to monovalent vaccines. Notably, the vaccine retained neutralization against phylogenetically distant coronaviruses, including bat-derived BANA-52, BANA-103, and hCoV-229E from the α genus, suggesting its capacity to overcome the phenomenon of “original antigenic sin.” Thus, this modular platform offers a rapid-response framework for addressing coronavirus evolution and cross-species transmission. Its design principles—targeting conserved epitopes and employing adaptive antigen presentation—provide a strategic blueprint for developing broad-spectrum vaccines against other rapidly evolving pathogens.

While the observed immunogenicity and breadth of neutralization in this study are promising, we recognize a significant limitation: the lack of *in vivo* challenge data to directly demonstrate protective efficacy. Conclusive evidence of protection necessitates evaluation in suitable animal models following live virus challenge. Our decision to postpone such experiments was informed by the specific interdisciplinary challenges associated with developing a vaccine targeting multiple coronavirus genera. Conducting cohesive challenge studies across phylogenetically diverse pathogens, such as SARS-CoV-2, MERS-CoV, and SARS-CoV-1, would require simultaneous access to high-level biocontainment facilities and is further complicated by the limited availability of well-characterized live virus strains for certain species, such as bat coronaviruses. Additionally, a universal animal model that is equally susceptible to infection by coronaviruses utilizing distinct entry receptors (ACE2, DPP4, and APN) is currently unavailable. In consideration of these constraints, we employed a comprehensive suite of rigorous *in vitro* surrogate assays that are established correlates of protection. The extensive pseudovirus neutralization observed across a diverse array of spike proteins, coupled with the elicitation of polyfunctional T-cell responses, offers compelling evidence for the functional efficacy and potential breadth of immunity induced by our nanoparticle platform. These findings constitute a crucial foundational step, establishing a robust rationale for future investment in the complex *in vivo* studies necessary to advance this vaccine strategy.

## MATERIALS AND METHODS

### Cell culture and plasmid construction

HEK293T cells, purchased from the American Type Culture Collection, were cultured in Dulbecco’s modified Eagle medium (DMEM, Gibco, Cat# C11995500), supplemented with 25 mM d-glucose, 1 mM sodium pyruvate, 1× non-essential amino acids, 10% (vol/vol) heat-inactivated fetal bovine serum (FBS, LONSERA, Cat# S711-001S), and 1% penicillin-streptomycin (Servicebio, Cat# G4003) at 37°C in a humidified atmosphere containing 5% CO_2_. The hACE2-HEK293T cell line was derived from the parental HEK293T cells through stable expression of human angiotensin-converting enzyme 2 (hACE2), achieved by lentiviral transduction with a vector encoding the full-length hACE2 gene. FreeStyle 293F cells were maintained in Union 293 medium (Union, Cat# UP1000), supplemented with 1% penicillin-streptomycin, at 37°C in an 8% CO_2_ environment with continuous shaking at 120 rpm.

The I53-DN5A was optimized, synthesized (Sangon Biotech), and cloned into the pcDNA3.1(+) vector (Life Technologies), with a C-terminal His_6_ tag. The RBD-HRC-DN5B plasmids, containing each of the N-glycosylation modified RBD (SARS-CoV-2 prototypic and BA.5 S-RBD (residues 333–527, with R357N and P521N mutations), MERS-CoV S-RBD (residues 381–588, with V403N and T579N mutations), SARS S-RBD (residues 321–512, K344N and P507N mutations)), an HRC domain derived from SARS-CoV-2 S and I53-DN5B, were optimized, synthesized, and cloned into the pLenti-puro vector, with an N-terminal CD5 signal peptide and a C-terminal His_6_ tag.

### Stably recombinant protein-expressing 293F cell line screening

A three-plasmid system was employed to co-transfect HEK293T cells with PSPAX2, PMD2.G, and the target plasmid at a ratio of 4:3:1 for lentivirus packaging. Following transient transfection, the cells were cultured for 72 h and then centrifuged at 12,000 × *g* for 5 min. The supernatant was collected into a sterile 2 mL centrifuge tube and stored at −80°C for subsequent use. Next, 30 mL of Expi293F cells were adjusted to a density of 1 × 10^6^ cells/mL, and 1 mL of thawed lentivirus was added to the culture. The mixture was incubated in a shaker incubator maintained at 37°C, 8% CO_2_, and 120 rpm. After 72 h, the cells were centrifuged at 800 × *g* for 5 min, and the pellet was resuspended in fresh complete medium supplemented with puromycin, adjusting the cell concentration to approximately 2 × 10^6^ cells/mL. Following approximately 2–3 weeks of continuous selection, the expression of the recombinant protein was assessed by Western blot analysis.

### Expression and purification of recombinant proteins

I53-DN5A was expressed in BL21 *Escherichia coli*. The cells were harvested by centrifugation, and the resulting pellet was resuspended in buffer A (20 mM Tris, 300 mM NaCl, pH 7.4) and lysed using a low-temperature, high-pressure continuous-flow cell disruptor until the solution became clear. The lysate was then centrifuged at 4°C and 12,000 × *g* for 10 min to separate the supernatant, which was subsequently filtered through a vacuum filtration system equipped with a 0.22 µm membrane for sterilization. RBD-HRC-DN5B was expressed using stably transfected cell lines and secreted into the supernatant. A tangential flow filtration system was used to exchange the supernatant into a buffer containing 20 mM Tris, 300 mM NaCl, pH 7.4. All proteins were purified using an Ni-NTA column followed by size-exclusion chromatography on a Superdex 200 gel filtration column (GE Healthcare), as previously described (31). The concentration of purified antigen proteins was quantified by measuring the absorbance at 280 nm (A280) using a microvolume UV-Vis spectrophotometer (NanoDrop 2000). The instrument was calibrated and blanked with the specific elution buffer used for the respective protein. Each sample was applied and measured three times to ensure technical reproducibility, and the mean *A*_280_ value was used for subsequent calculation. The protein concentration was determined using the formula: Concentration (mg/mL) = (*A*_280_/ε) × Molecular wt, where *ε* is the theoretical molar extinction coefficient calculated from the amino acid sequence. The purified proteins were stored in a buffer containing 20 mM Tris (pH 7.2) and 200 mM NaCl for further use.

### Extracellular assembly and purification of the RBD-HRC-TRIMER nanoparticles

To assemble the two components, RBD-HRC-DN5B and DN5A, each was introduced into a 10 kDa ultrafiltration tube and diluted to a concentration of 1 mg/mL using an assembly buffer composed of 50 mM HEPES at pH 7.4 and 300 mM NaCl. Subsequently, the chimeric single-antigen protein nanoparticles were combined at a molar ratio of 1:1 for DN5A to DN5B at room temperature. For the chimeric two-antigen protein nanoparticles, a molar ratio of 2:1:1 for DN5A to DN5B was used, and for the chimeric three-antigen protein nanoparticles, a ratio of 3:1:1 was employed, with mixing conducted for 1 h on a rotary mixer. Following extracellular assembly and incubation, the samples were centrifuged at 17,000 × *g* for 10 min at 4°C to eliminate aggregated proteins. The samples were then further purified via gel filtration chromatography using a Superose 6 Increase 10/300 GL column (Cytiva, Cat# 29091596), pre-equilibrated with PBS. The elution fractions containing the assembled nanoparticle immunogens were collected, combined, concentrated using a 10 kDa ultrafiltration tube, and sterilized. The samples underwent centrifugation at 3,900 × *g* for 10 min at 4°C utilizing a centrifugal filter with a pore size of 0.22 μm (Corning, Cat# 8160). Subsequently, all purified immunogens were sterilized using the same 0.22 μm pore size centrifugal filter and stored at 4°C.

The nanoparticle size and the Zeta potential of the cage-like structure within the assembly buffer were assessed using a Nano-ZS90 laser particle size analyzer (Malvern Instruments Ltd.). A sample with a concentration of 0.5 mg/mL was applied onto a carbon-coated grid that had been freshly glow-discharged, followed by rinsing with a 2% phosphotungstic acid (PTA) solution for 5 min. After air drying, the grid was examined using a transmission electron microscope operating at 120 kV.

### Mice immunization with the chimeric protein nanoparticle

Forty healthy female BALB/c mice, aged 6–8 weeks, were purchased from Henan Province Laboratory Animal Management Committee of China for this study. The mice were randomly assigned to eight experimental groups, with five mice per group. Each mouse received a subcutaneous injection of 0.2 mL of solution on the dorsal surface, corresponding to an immunization dose of 10 µg per mouse. Throughout the study, all groups had unrestricted access to food and water. The immunization protocol consisted of two phases: primary immunization on day 1 and a booster immunization on day 21. Blood samples were collected from the tail vein on days 14, 28, and 35 following the primary immunization. The collected blood was first incubated at 37°C for 30 min, then kept at 4°C for 4 h, and finally centrifuged at 3,000 rpm for 10 min at 4°C. The resulting serum was separated, aliquoted, and stored at −80°C for further analysis.

### Production of coronavirus spike-pseudotyped viruses

Retroviruses pseudotyped with coronavirus spike proteins were generated as previously described. In brief, pseudoviruses were produced by co-transfecting HEK293T cells with a plasmid containing an Env-defective, luciferase-expressing HIV-1 genome (pNL4-3. luc.RE) and a plasmid encoding the coronavirus spike protein. The spike proteins used in this study included the SARS-CoV-2 prototype and its variants (D614G, BA.5, and JN.1), two SARS-CoV-2-related bat coronaviruses (BANAL-52 and BANAL-103), SARS-CoV, MERS-CoV, and HCoV-229E. At 72 h post-transfection, the pseudoviruses were harvested, and the supernatant was collected. The supernatant was then clarified by centrifugation at 12,000 × *g* to remove cellular debris and stored at −80°C for later use.

### Serum enzyme-linked immunosorbent assay (ELISA) binding assay

To evaluate the antibodies induced in the serum of immunized mice, two purified RBD monomers and four purified RBD-HRC-DN5B proteins were used as antigens. In brief, recombinant antigens (200 ng per well) were immobilized onto 96-well microplates using PBS and incubated overnight at 4°C. The following day, the plates were washed and blocked as previously described. For the determination of endpoint titers, heat-inactivated serum samples from immunized mice were serially diluted at ratios of 1:10^2^, 1:10^3^, and 1: 10^4^ with PBS and added to the plates. For the comparative analysis of antigen-specific IgG and subclass levels presented in Fig. 2B through J and Fig. S2A through R, serum samples were tested at a single dilution of 1:100. After a 1-h incubation at 37°C, the plates were washed five times with PBST and then incubated with a 1:20,000 dilution of HRP-conjugated goat anti-mouse IgG secondary antibody in PBST containing 2% BSA for 45 min at 37°C. Following five additional washes with PBST, the plates were developed with TMB substrate at 37°C for 10 min, and the reaction was stopped using a stop solution. Absorbance at 450 nm (*A*_450_) was measured using a Feyond A300 multifunctional microplate reader, with a positive/negative (P/N) ratio greater than 2 considered as the threshold for a positive result. To further enhance the robustness of quantitative analysis for the data in Fig. 2B through J and Fig. S2A through R, the area under the curve (AUC) was calculated for each sample by plotting the *A*_450_ (AU) values against the logarithm of the dilution factor, and integrating data from the three time points (14, 28, and 35 days post-primary immunization).

The concentrations of specific IgG1 and IgG2a in the serum were determined following the same procedure. In brief, Goat anti-Mouse IgG1 Cross-Adsorbed Secondary Antibody, HRP (Thermo, Cat# A10551), and Goat anti-Mouse IgG2a Secondary Antibody, HRP (Thermo, Cat# A10685), were diluted at 1:10,000 according to the previously described protocol.

### Spleen lymphocyte isolation and flow cytometric analysis

Spleens were collected from immunized mice and homogenized using a 70 µm filter. Lymphocytes were then isolated using a commercial kit (Solarbio, Cat# P8860). For T-cell analysis, single-cell suspensions from the spleen were stained at 4°C with 7-AAD-Percp-Cy5.5 (BD Pharmingen, Cat# 559925), anti-CD45-FITC (BD Pharmingen, Cat# 553079), anti-CD3-PE-Cy7 (BD Pharmingen, Cat# 552774), and anti-CD21-PE (BD Pharmingen, Cat# 568014), followed by flow cytometric analysis and data processing using FlowJo software. For analysis of CD4^+^ and CD8^+^ T cells, single-cell suspensions from the spleen were similarly stained at 4°C with 7-AAD-Percp-Cy5.5, anti-CD3-PE-Cy7, anti-CD4-FITC (BD Pharmingen, Cat# 557307), and anti-CD8-PE (BD Pharmingen, Cat# 553032), followed by flow cytometric analysis and subsequent data analysis using FlowJo software.

### Serum neutralization assay for coronavirus spike-pseudotyped viruses

For the pseudovirus-based neutralization assay, inactivated serum obtained from immunized mice was diluted 1:100 in PBS and then mixed with an equal volume of pre-titrated pseudovirus. Following a 1-h incubation at 37°C, the serum-pseudovirus mixture was added to a 96-well plate pre-seeded with the indicated cells. As negative controls, cells were infected with pseudovirus lacking the coronavirus spike protein, while positive controls consisted of cells infected only with spike-pseudotyped viruses. After an additional 48-h incubation, the cells were washed once with PBS, lysed, and firefly luciferase activity was measured (Allsheng Feyond-A300) as an indicator of viral entry, following a previously described protocol (62). Neutralization efficacy was quantified as a percentage of neutralization based on luciferase activity, calculated using the formula: % Neutralization = [1 − (RLU_sample − RLU_cell control)/(RLU_virus control − RLU_cell control)] × 100%. Data were analyzed using GraphPad Prism version 8.0 software.

### Quantification and statistical analysis

GraphPad Prism version 8.0 was used for statistical analysis. Experimental results are presented as mean ± standard deviation (mean ± SD). The independent samples *t*-test was employed to evaluate differences between the two groups. A *P* value less than 0.05 was considered statistically significant, where * indicates *P* < 0.05, ** denotes *P* < 0.01, and *** represents *P* < 0.001.

## Data Availability

The data sets used during the current study are available from the corresponding authors upon reasonable request.

## SUPPLEMENTAL MATERIAL

Fig. S1 spectrum.03797-25-s0001.tif Production and characterization of RBD-HRC nanoparticle vaccines.

Fig. S2 spectrum.03797-25-s0002.tif Serum levels of specific IgG1 and IgG2a antibodies in immunized mice.

Supplemental material spectrum.03797-25-s0003.docx Supplemental figure legends.

ASM does not own the copyrights to Supplemental Material that may be linked to, or accessed through, an article. The authors have granted ASM a non-exclusive, world-wide license to publish the Supplemental Material files. Please contact the corresponding author directly for reuse.
