# Supplementary figures and images for: A self-assembled nanoparticle vaccine displaying chimeric and trimeric RBD-HRC elicits broad-spectrum neutralizing antibodies against multiple coronaviruses
Source: Microbiol Spectr. 2026 Mar 30;14(5):e03797-25. doi: 10.1128/spectrum.03797-25 (PMC13141874; doi:10.1128/spectrum.03797-25)

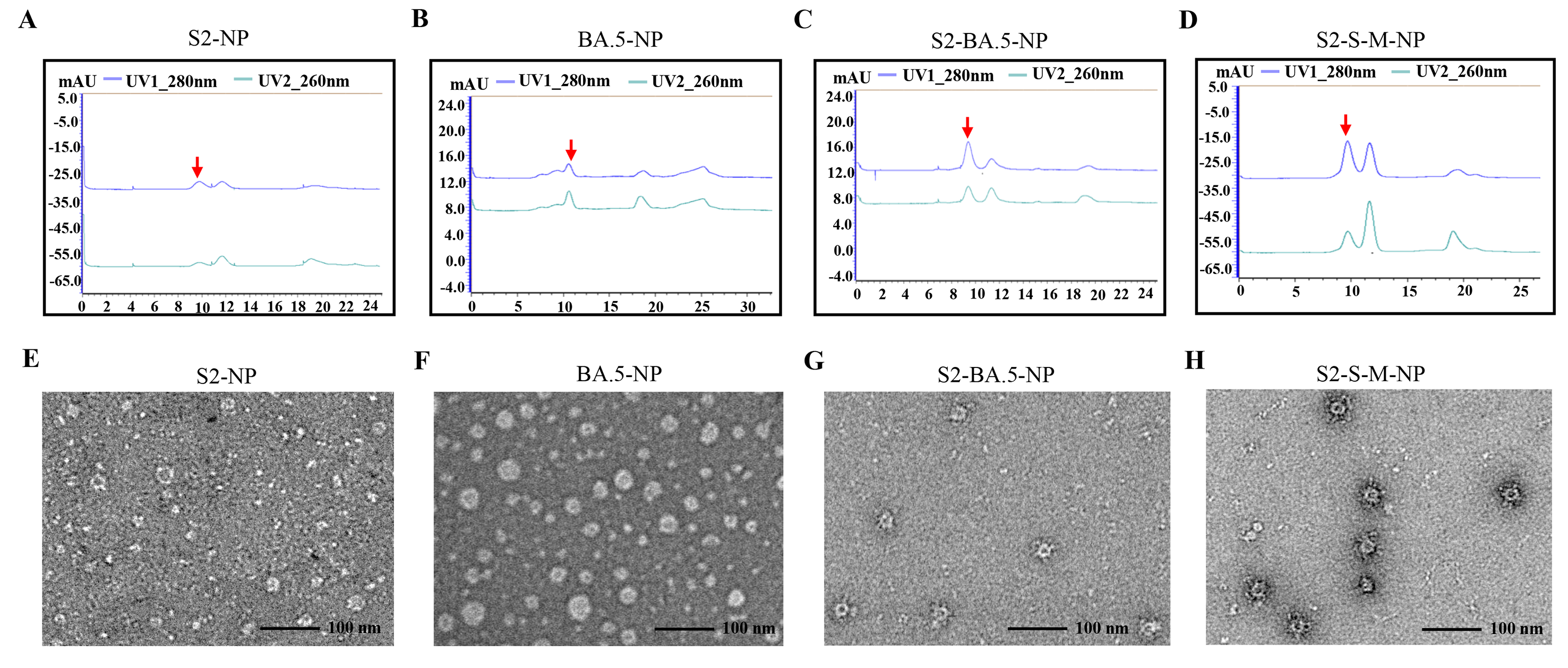

Supplement: Fig. S1 — Production and characterization of RBD-HRC nanoparticle vaccines. [file spectrum.03797-25-s0001.tif]

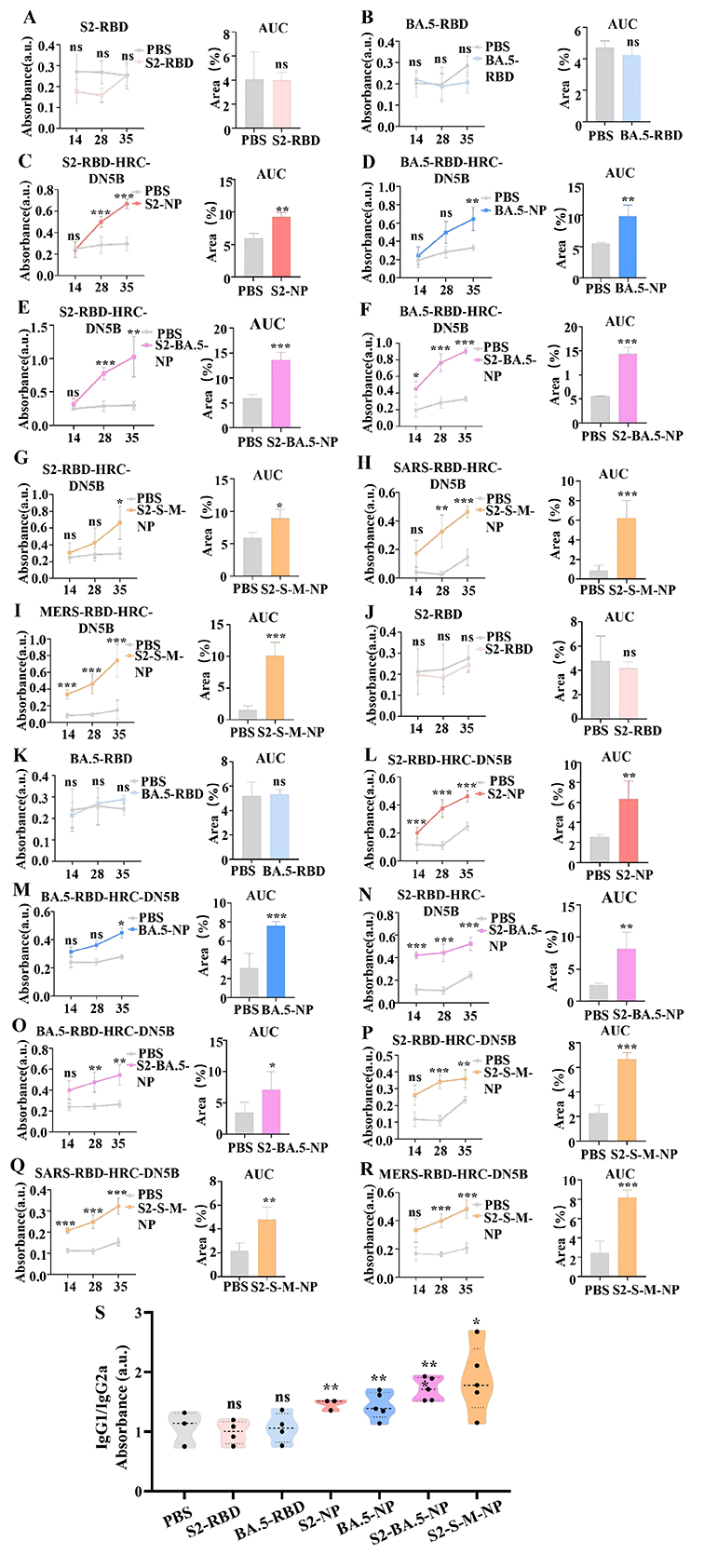

Supplement: Fig. S2 — Serum levels of specific IgG1 and IgG2a antibodies in immunized mice. [file spectrum.03797-25-s0002.tif]
