# Supplementary material for: A self-assembled nanoparticle vaccine displaying chimeric and trimeric RBD-HRC elicits broad-spectrum neutralizing antibodies against multiple coronaviruses
Source: Microbiol Spectr. 2026 Mar 30;14(5):e03797-25. doi: 10.1128/spectrum.03797-25 (PMC13141874; doi:10.1128/spectrum.03797-25)
Supplement: Supplemental material — Supplemental figure legends. [file spectrum.03797-25-s0003.docx]

**A Self-Assembled Nanoparticle-Displayed Chimeric and Trimeric RBD-HRC Vaccine Elicits Broad-Spectrum Neutralizing Antibodies Against Multiple Coronaviruses**

Didi Wan^1#^, Lili Li^1#^, He Feng^1^, Mengyu Wang^1^, Shuo Jia^1^, Pengyu Zhang^1^, Jiaxu Wang^3^, Junxia Yang^1^, Yijie Zhang^1^, Boya Ji^1^, Yushun Wan^4^, zhengkun, Xie^5^, Jian Shang^1,2^*

^1^BGI College & Henan Institute of Medical and Pharmaceutical Sciences, Zhengzhou University, Zhengzhou, 450052, Henan, China.

^2^State Key Laboratory of Metabolic Dysregulation & Prevention and Treatment of Esophageal Cancer, School of Life Sciences, Zhengzhou University, Zhengzhou, 450001, Henan, China

^3^College of Life Sciences, Henan Normal University, Xinxiang, 453007, Henan, China

^4^College of Basic Medicine, Chongqing Medical University, 400016, Chongqing, China

^5^College of Chemistry, Zhengzhou University, Zhengzhou, 450001, Henan, China

^#^These authors contribute equally to this work

*Corresponding author: Jian Shang (jianshang0210@zzu.edu.cn); No. 40 Daxue Road, Zhengzhou, 450052, P. R. China. Tel: +86-18700025681

The authors declare no conflict of interest

**Supplemental figure legends**

Fig. S1. Production and characterization of RBD-HRC nanoparticle vaccines. (A–D) In vitro self-assembly and separation of nanoparticle vaccines. The peaks indicated by the red arrows were identified using SDS-PAGE and subsequently concentrated for further use. (E–H) Each purified nanoparticle vaccine was analyzed by negative-staining electron microscopy. S2-NP: SARS-CoV-2 RBD-HRC nanoparticles; BA.5-NP: Omicron BA.5 RBD-HRC nanoparticles; S2-BA.5-NP: A divalent nanoparticle vaccine containing both SARS-CoV-2 and BA.5 RBD-HRCs; S2-S-M-NP: A trivalent nanoparticle vaccine containing SARS-CoV-2, SARS, and MERS RBD-HRCs.

Fig. S2. Serum levels of specific IgG1 and IgG2a antibodies in immunized mice. (A-I) Serum specific IgG1 antibody levels in mouse sera were evaluated by indirect ELISA at 14, 28, and 35 days after primary immunization. (J-R) Serum specific IgG2a antibody levels in mouse sera were similarly assessed using indirect ELISA at 14, 28, and 35 days post-primary immunization. Each panel in (A-I) and (J-R) contains two subplots: the left subplot shows AU obtained at a serum dilution of 1:100 for each time point, and the right subplot presents the AUC values integrated from the AU data of the three time points. (S) The IgG1/IgG2a ratios for the different immunization groups were calculated based on the specific antibody levels presented in panels A and B. S2-NP: SARS-CoV-2 RBD-HRC nanoparticles; BA.5-NP: Omicron BA.5 RBD-HRC nanoparticles; S2-BA.5-NP: A divalent nanoparticle vaccine containing both SARS-CoV-2 and BA.5 RBD-HRCs; S2-S-M-NP: A trivalent nanoparticle vaccine containing SARS-CoV-2, SARS, and MERS RBD-HRCs. All data are shown as mean ± SEM (n=5). Statistical significance was determined using a t-test, with significance levels indicated as follows: **P*<0.05, ***P*<0.01, ****P*<0.001.
